# Inhibition of expression of the circadian clock gene *Period* causes metabolic abnormalities including repression of glycometabolism in *Bombyx mori* cells

**DOI:** 10.1038/srep46258

**Published:** 2017-04-10

**Authors:** Hui Tao, Xue Li, Jian-Feng Qiu, Wen-Zhao Cui, Yang-Hu Sima, Shi-Qing Xu

**Affiliations:** 1 School of Biology and Basic Medical Sciences, Medical College, Soochow University, Suzhou 215123, China; 2 Institute of Agricultural Biotechnology & Ecology (IABE), Soochow University, Suzhou 215123, China; 3 National Engineering Laboratory for Modern Silk (NEAER), Soochow University, Suzhou 215123, China

## Abstract

Abnormalities in the circadian clock system are known to affect the body’s metabolic functions, though the molecular mechanisms responsible remain uncertain. In this study, we achieved continuous knockdown of *B. mori Period (BmPer)* gene expression in the *B. mori* ovary cell line (BmN), and generated a Per-KD *B. mori* model with developmental disorders including small individual cells and slow growth. We conducted cell metabolomics assays by gas chromatography/liquid chromatography-mass spectrometry and showed that knockdown of *BmPer* gene expression resulted in significant inhibition of glycometabolism. Amino acids that used glucose metabolites as a source were also down-regulated, while lipid metabolism and nucleotide metabolism were significantly up-regulated. Metabolite correlation analysis showed that pyruvate and lactate were closely related to glycometabolism, as well as to metabolites such as aspartate, alanine, and xanthine in other pathways. Further validation experiments showed that the activities of the key enzymes of glucose metabolism, hexokinase, phosphofructokinase, and citrate synthase, were significantly decreased and transcription of their encoding genes, as well as that of pyruvate kinase, were also significantly down-regulated. We concluded that inhibition of the circadian clock gene *BmPer* repressed glycometabolism, and may be associated with changes in cellular amino acid metabolism, and in cell growth and development.

Metabolic regulation is the most important output of circadian clock signals^1^ across a wide range of species from *Chlorococcus*^2^ to humans^3^, though the specific molecular mechanisms involved remain unclear^1^,^3^,^4^. Studies have shown that important metabolic processes, such as glycometabolism and cholesterol metabolism, are controlled by the circadian clock^5^. Circadian clock disorders thus lead not only to sleep and mental disorders, but also to severe metabolic diseases^6^, including obesity^7^,^8^, diabetes^9^, hypertension and other cardiovascular diseases^10^,^11^, and may even be associated with accelerated aging^12^ and tumor development^13^.

The *period* protein (PER) is a core component of the circadian clock. It includes a PAS (Per-Art-Ser) domain^14^, and plays a role in signal conduction in the negative feedback loop involved in circadian clock core oscillations. The mammalian PER protein is able to bind the CLOCK/BMAL protein dimer to regulate the expression of downstream genes^15^. Recent studies have found that PER not only plays a role in regulating gene expression, but also has an important impact on metabolism; however, the results of studies have been inconsistent or even contradictory. Feillet *et al*.^16^ reported no significant changes in liver glycogen content in *mPer2* mutant mice, while later studies found that silencing the expression of *mPer2* led to changes in blood sugar, liver glycogen, and insulin^17^,^18^,^19^. *mPer1* knockout caused increased blood pressure in mice^20^, while simultaneous knockout of *mPer1*/*mPer2* or of *mPer2* alone both led to lower triglyceride levels in mouse liver^21^,^22^. Similarly, the results of two studies on corticosterone metabolism in *mPer* mutant mice produced conflicting results in terms of metabolic damage and enhancement^23^,^24^.

Biological metabolism is a complex regulatory process, largely controlled by endocrine hormones^25^, and subject to interactions between different metabolic pathways^26^. However, systematic studies of the correlation between the circadian clock and metabolism are lacking, and most studies have only investigated single indicators, such as blood glucose, liver glycogen, and triglycerides, and have not fully assessed changes in all metabolites. An overall metabolomics analysis is therefore required. However, *Per* genes in mammals, such as mice and humans, include multiple gene subtypes, and the gene expression levels of mouse *mPer1, mPer2*, and *mPer3* differ among different tissues and developmental stages, along with differences in the functions of the protein products^27^,^28^,^29^,^30^. No studies to date have investigated the metabolic effects of knockdown/out of all *mPer* genes simultaneously. It may therefore be possible to obtain a clearer understanding of the metabolic effect of the circadian clock by selecting a model animal with only one type of *Per* gene, thus avoiding interactions among multiple genes. We therefore investigated this relationship in the silkworm *Bombyx mori*, which only has one type of PER protein (Figure S1). We further knocked down PER protein expression continuously in a cell line *in vitro*, to eliminate any potential endocrine effects. We performed metabolomic analysis in *B. mori* using gas chromatography/liquid chromatography-mass spectrometry (GC/LC-MS), to evaluate the effect of the peripheral circadian clock on cellular metabolism, and to investigate the relationship between repression of glycometabolism and other metabolic pathway abnormalities caused by changes in the circadian clock system, by detailed evaluation of changes in glucose metabolic pathways.

## Results

### Effect of knockdown of *BmPer* gene expression on circadian clock system and growth of BmN cells

**Figure 1:**
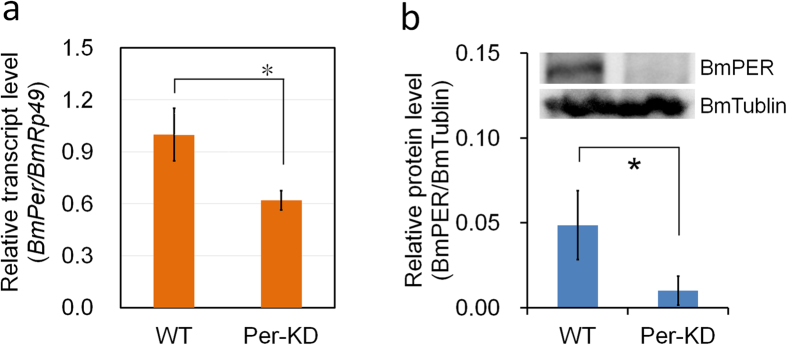
Knockdown efficiency of *BmPer* gene. (**a**) *BmPer* transcripts were analyzed by qRT-PCR with *BmRp49* as an internal control. (**b**) BmPER protein levels determined by western blot with BmTublin protein as an internal control. WT, wild-type BmN cells; Per-KD, *BmPer* knockdown BmN cells. *p ≤ 0.05 (repeated three times).

Screening revealed that *BmPer* gene expression was continuously knocked down in *B. mori* ovary cells transfected with a recombinant vector expressing the interference sequence of the *BmPer* gene (Per-KD). *BmPer* gene transcription in Per-KD cells, determined by quantitative reverse transcription-polymerase chain reaction (qRT-PCR), was downregulated by about 40% compared with wild type (WT) cells (Fig. 1a), while BmPER protein levels were significantly reduced by about 80%, according to western blotting (Fig. 1b). *BmPer* gene knockdown thus significantly reduced both gene and protein expression of *Per*. We investigated transcription of the genes encoding proteins involved in circadian clock oscillation, BmCRY1 (Cryptochrome1), BmCRY2 (Cryptochrome2), BmTIM (Timeless), BmCLK (Clock), and BmCYC (Cycle), and showed that rhythms in expression levels of all five genes were decreased or even absent after synchronization with dexamethasone (Dex), with expression levels of *BmTim* and *BmClk* genes being significantly down-regulated (Figure S2). These results indicated that continuous down-regulation of *BmPer* gene transcription and protein translation in Per-KD cells further disrupted the transcription of genes encoding the circadian oscillators, suggesting that circadian signal outputs may be affected in Per-KD cells.

**Figure 2:**
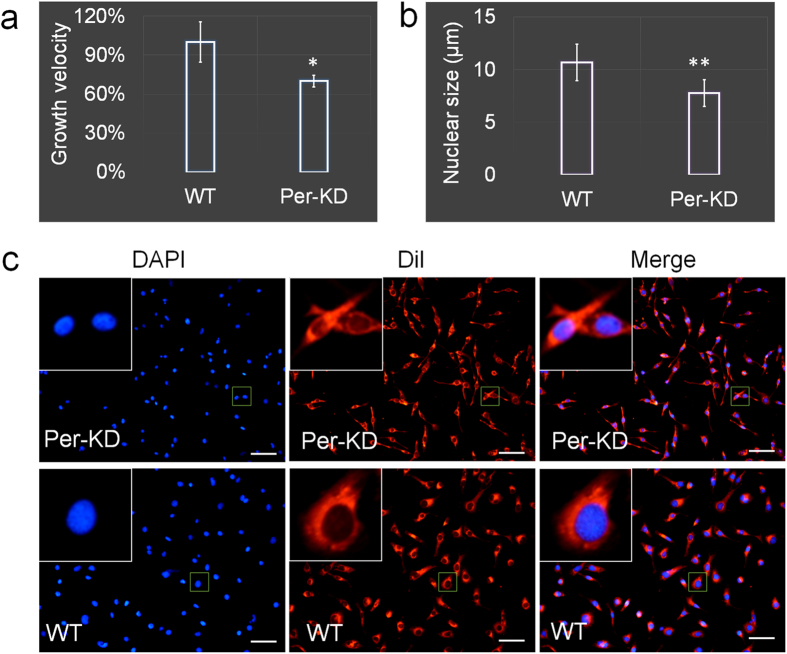
Effect of *BmPer* gene knockdown on BmN cell growth. (**a**) Cell growth velocity measured by MTT method. (**b**) Cells were transferred to a coverslip when they reached 70% confluence. After the cells were completely adherent (at least 12 h) the diameters of the nuclei were measured (average of the longest and shortest diameters). (**c**) Cell nuclei stained with 4′-6-diamidino-2-phenylindole (DAPI) and cell membranes stained with Dil (1,1′-dioctadecyl-3,3,3′,3′-tetramethylindocarbocyanine perchlorate) (showing cell morphology). WT, wild-type BmN cells; Per-KD, *BmPer* knockdown BmN cells. *p ≤ 0.05 and **p ≤ 0.01 (n = 3 plates, observations and statistical analyses were repeated at least five times). Bar = 50 μm.

Per-KD cells showed distinctly different phenotypic traits from WT cells. Cell growth rate measured by MTT showed that Per-KD cells grew slower than WT cells; the growth velocity of Per-KD cells was decreased by about 30% within 4 h of measurement (Fig. 2a). Staining of cell membranes and nuclei also revealed that Per-KD cells were smaller than WT cells (Fig. 2b), and the average diameter of WT nuclei stained with DAPI was 10.5 μm, compared with 7 μm in Per-KD nuclei (p ≤ 0.05) (Fig. 2b,c).

### Metabolomic changes in Per-KD cells

The phenotypic differences indicated that *BmPer* knockdown had a negative effect on the growth and development of Per-KD cells, suggesting potential effects on the metabolism responsible for maintaining cell growth and development. We therefore investigated the metabolomic changes by GC-MS and LC-MS. The ion current chromatograms of eight repeated assays are shown in Figures S3 and S4.

**Figure 3:**
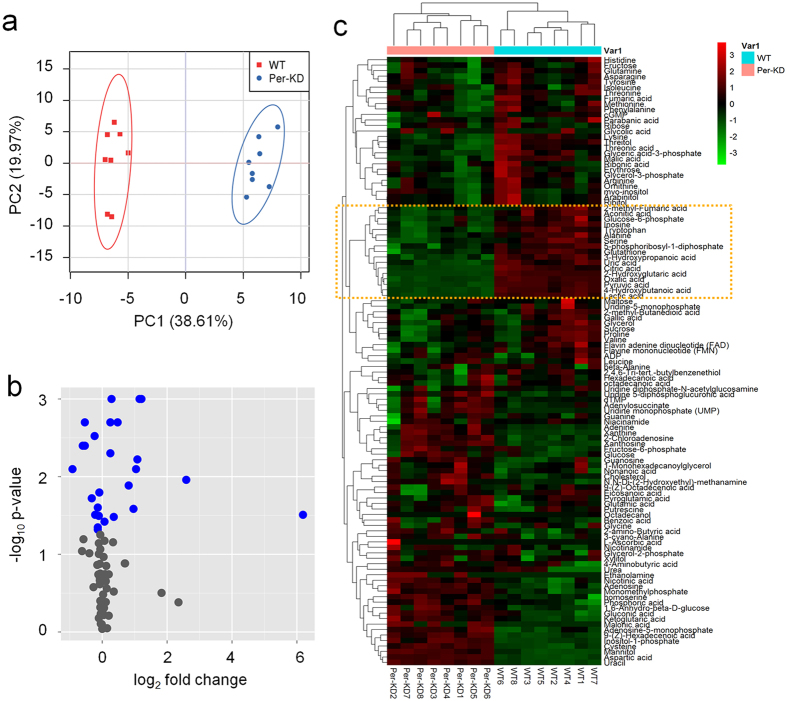
LC/GC-MS assay based metabolomics. PCA (**a**), volcano plot (**b**), and heat map (**c**). In the volcano plot, differential metabolites (blue) and non-differential substances (gray) were screened out under the conditions of fold change ≥1 and p ≤ 0.05. WT, wild-type BmN cells; Per-KD, *BmPer* knockdown BmN cells. WT 1–8 and Per-KD 1–8 on the x axis in (**c**) indicate eight repeated assays.

The original data were processed and analyzed using XCMS software (www.bioconductor.org/)^31^. A total of 112 differential metabolites were detected and identified in Per-KD cells, using WT BmN cells as a control. Prinicipal components analysis (PCA) of differential metabolites was carried out to obtain a PCA score plot (Fig. 3a). PCA simulation identified two principal components that explained 58.58% of the model; PC1 could explain 38.61% of the data model, and PC2 could explain 19.97%. These results indicated that the substances detected by metabolomic method could clearly distinguish between Per-KD BmN and WT cells.

Partial least squares discriminant analysis (PLS-DA) score plots showed that supervised multivariate analysis revealed more obvious differences between the metabolites in the two kinds of cells, indicating a significant difference in metabolic components between Per-KD and WT cells (Figure S5a). A volcano plot (Fig. 3b) showed the difference in metabolites between the two kinds of cells, calculated from data for all tested substances. Cluster analysis of all the samples further revealed that the repeat results for Per-KD cells and WT cells, respectively, clustered together, indicating a significant difference in metabolism between the two kind of cells (Figure S5b).

All the detected metabolites were analyzed by heat mapping. Some metabolites differed significantly between Per-KD and WT cells, in stable and consistent manners within groups (Fig. 3c). Glucose metabolites such as pyruvate, lactate, and glucose-6-phosphate produced in the glycolytic pathway, and amino acids such as alanine, serine, and tyrosine produced from glycolytic intermediates were abundant in WT cells but low in Per-KD cells (Fig. 3c, orange box), suggesting that knockdown of *BmPer* gene expression had significant effects on glucose and amino acid metabolism in Per-KD cells.

We further screened for differential metabolites between Per-KD and WT cells using the PLS-DA model first principal component variable importance in projection (VIP) value ≥1 and *t*-test (p ≤ 0.05) as thresholds for GC-MS assay results. Standard tests were carried out for a certain number of substances, and PLS-DA model first principal component VIP ≥1, p (corr) ≥0.08 of the S-plot and p ≤ 0.05 for *t*-tests were chosen as thresholds to screen out differential metabolites from LC-MS assay results, while some substances were tested by secondary mass spectrometry. Differential metabolites are summarized in Tables 1 and 2. Differential metabolites between the two types of cells identified by GC-MS were mainly amino acids, glucose metabolites, and organic acids, including fatty acids (Table 1), while differential metabolites identified by LC-MS secondary mass spectrometry were mainly nucleotides and their derivatives (Table 2).

**Figure 4:**
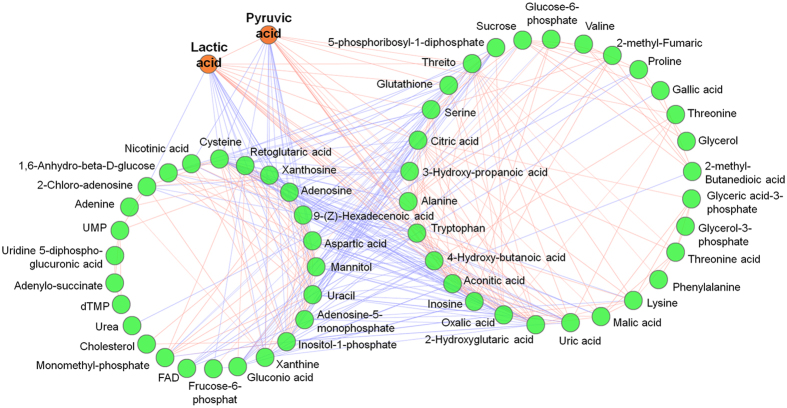
Correlation network diagram of differential metabolites. Metabolite correlation analysis was carried out using Pearson’s correlation coefficient, with the cor() function in the R language package (www.r-project.org). The Pearson’s correlation coefficient threshold was set as 0.8. Red lines represent positive correlations between substances, blue lines represent negative correlations between substances.

We further analyzed the screened out differential substances using a differential metabolite correlation network. As shown in Fig. 4, 59 differential metabolites constituted numerous edges and formed a complex topological network, indicating that the metabolites in Per-KD cells changed actively. According to the concept of a “clustering factor”^32^, the more correlations formed by a metabolite, the bigger its clustering factor, indicating that it may act as an important information link in the topological structure. Pyruvic acid and lactic acid constituted up to 25 and 24 edges in the network, respectively, indicating that these two metabolites played important central hub roles in the topological network, and potentially in the regulation of the entire cell metabolism.

**Figure 5:**
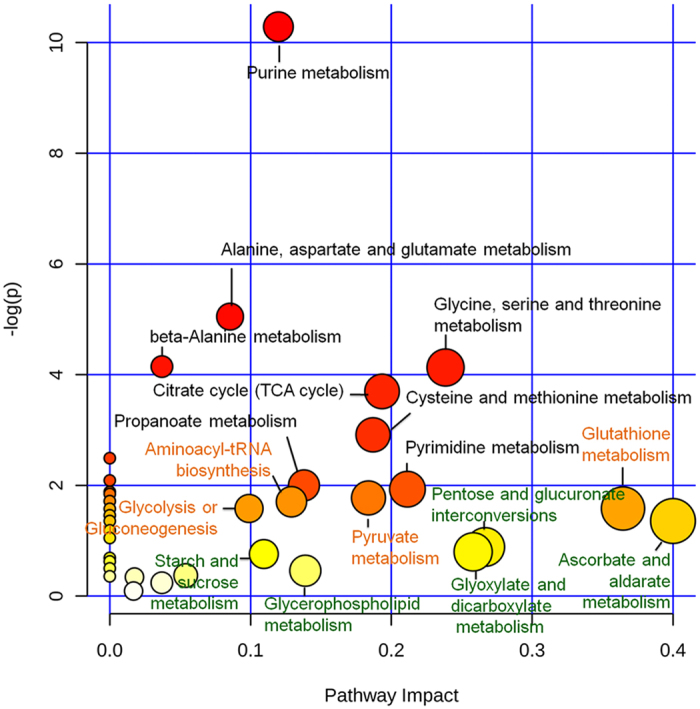
MetPA analysis of metabolic pathways. We analyzed the MetPA plot using the online software http://www.metaboanalyst.ca/, with default values as the analysis parameters. The deeper the red color of the metabolic pathway, the greater its -log (p) value, indicating a more significant difference. The darker the yellow color, the greater the impact of the pathway.

We carried out Metabolomics Pathway Analysis (MetPA) analysis of the metabolic pathways of the differential metabolites based on visual analysis^33^ to clarify the overall statuses of the differential metabolites in the two kinds of cells. There were extensive changes in the activities of pathways including nucleotide, amino acid, carbohydrate, and lipid metabolism, and synthesis of secondary metabolites in Per-KD cells, indicating that the differential metabolites impacted on the overall metabolism in the cells (Fig. 5). The metabolites with most significant impacts were involved in purine metabolism (nucleotide metabolic system), aspartate, alanine, glycine, and glutamate metabolism (amino acid metabolic system), and glycolysis and the tricarboxylic acid (TCA) cycle (glucose metabolism). The effect on lipid metabolism in Per-KD cells was relatively weak, and differential metabolites mainly affected the cholesterol and steroid synthesis pathways. These affected pathways provided clues to guide subsequent study of the effects of BmPER.

### Glucose metabolism disorder in Per-KD cells

Per-KD cells grew more slowly and were smaller than WT cells (Fig. 2). The metabolomic results (Fig. 4) further suggested that pyruvate and lactate may be important links among differential metabolites, while the activity of the glycolytic pathway was significantly affected in Per-KD cells (Fig. 5). We therefore speculated that energy metabolism may have been affected by *BmPer* knockdown in Per-KD cells.

**Figure 6:**
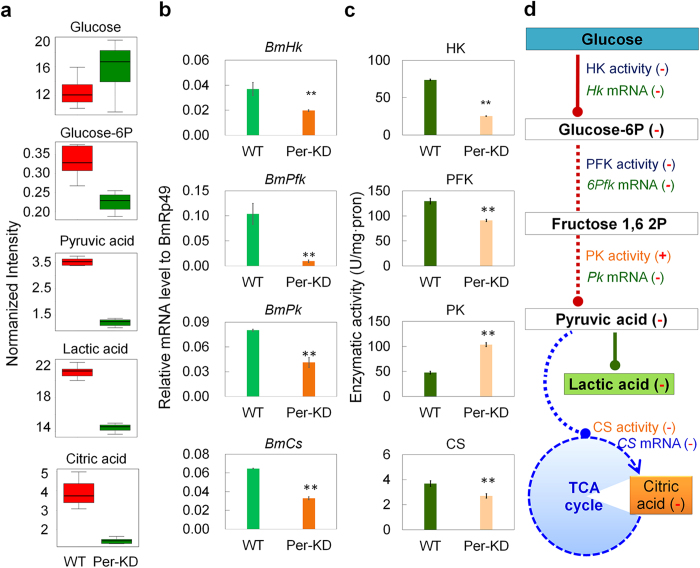
Effect of *BmPer* gene knockdown on glucose metabolism in BmN cells. (**a**) Changes in contents of main substrates and products, based on metabolomic measurements. (**b**) Transcription levels of the main rate-limiting enzyme-encoding genes. (**c**) Activities of the main rate-limiting enzymes. (**d**) Main affected glucose metabolic pathways. Per-KD, *BmPer* knockdown BmN cells; WT, wild-type BmN cells; HK, hexokinase; PFK, phosphofructokinase; PK, pyruvate kinase; CS, citrate synthase; +, increased enzymatic activity; −, decreased content or enzyme activity, or down-regulated transcription of coding gene. *p ≤ 0.05 and **p ≤ 0.01.

We investigated the effect of *Per* gene knockdown on glucose metabolism by first selecting important differential metabolites in the glucose metabolic system from the metabolomic assay results (Fig. 6a). Although there was sufficient glucose as a raw material of glycolysis in Per-KD cells, some products in the glycolysis process, especially products after the rate-limiting step, such as glucose-6 phosphate, pyruvate, and lactate, were decreased, indicating repression of the glycolytic pathway.

Furthermore, transcription levels of *BmHk(Hexokinase*), *BmPfk(Phosphofructokinase*), and *BmPk(Pyruvate kinase*), which encode the rate-limiting enzymes HK, PFK, and PK in the glycolytic pathway, were down-regulated by >40% in Per-KD cells compared with WT cells, with the transcript level of *BmPfk* reduced by 90% (Fig. 6b). The enzymatic activities of BmHK and BmPFK were also significantly reduced in Per-KD cells, consistent with down-regulation of transcript levels of their coding genes (Fig. 6c), thus explaining the mechanisms responsible for repression of the glycolytic pathway in Per-KD cells.

In contrast to the other rate-limiting enzymes, BmPK activity was significantly increased in Per-KD cells, even though transcription of its coding gene *BmPk* was down-regulated (Fig. 6c). Like pyruvate, the content of its anaerobic metabolite lactate was also reduced in Per-KD cells (Fig. 6a). Furthermore, the TCA cycle starting with pyruvate was also affected. The above metabolomic assays revealed a significant decrease in citrate content in Per-KD cells (Fig. 6a), which was the third largest change among all the metabolites (Table 1). The results for CS (*Citrate synthase*), the first rate-limiting enzyme in the TCA cycle, indicated that transcription of the gene encoding BmCS (Fig. 6b), as well as its enzymatic activity, were also significantly decreased (Fig. 6c). These results suggested that inhibition of the TCA cycle in Per-KD cells was caused by reduced pyruvate levels, in turn caused by decreased glycolysis and thus lack of feed into the TCA cycle, while the key enzyme of the first step of the TCA cycle was also inhibited.

These results suggest that glucose metabolism and glycometabolism-related pathways, including glycolysis and its further anaerobic and aerobic phases (TCA), were all significantly inhibited by *Per* gene knockdown.

## Discussion

The circadian clock has been reported to regulate glycometabolism in humans^34^ and other mammals^35^ containing polymorphic PER proteins. It is possible to estimate the time of the day in mammals based on the expression patterns of various substances^36^. The major clusters of circadian clock-regulated genes participate in principal functions in the liver, and many comprise rate-limiting steps in their respective pathways^37^. However, none of the metabolic changes investigated to date have been determined under conditions involving knockdown/out all of *mPer* genes simultaneously, and without the influence of animal endocrine hormones. We therefore chose to study the silkworm *B. mori*, which only expresses one PER protein, and to establish a cell line with continuous knockdown of BmPER protein expression. Metabolomic assays revealed that levels of pyruvate and lactate were significantly reduced in cultured Per-KD cells in the absence of any effect of endocrine hormones. Examination of the main rate-limiting steps in glycolysis and the TCA cycle confirmed that glucose metabolic processes were repressed in Per-KD cells (Fig. 6). Knockdown of BmPER levels in BmN cells thus induced consistent changes in the regulation of glucose metabolism, suggesting that lack of expression of this peripheral circadian oscillator core member caused significant changes in metabolic systems, including glucose metabolism.

Circadian control of physiology and behavior in mammals is driven by a master pacemaker located in the suprachiasmatic nucleus in the hypothalamus^38^,^39^,^40^. However, cell-autonomous clocks (peripheral clocks) have been found to be ubiquitous, and most cells in the body contain their own circadian clock^41^, as verified in cell lines ranging from mammals (mice)^42^ to lepidoptera species such as *Danaus plexippus*^43^. The results of the current study confirmed the existence of a peripheral clock in silkworm BmN cells^44^. Recent studies have shown that changes in feeding patterns may disrupt the peripheral clock in mice, leading to metabolic syndrome^45^,^46^, while a fat-metabolism disorder in *Drosophila* caused by food restriction was also regulated by the peripheral clock^47^. However, although individual animal cells have spontaneous rhythms, they are also regulated by the core cranial nerve core. We therefore examined the regulatory effect of the peripheral clock on cell metabolism in BmN cells *in vitro*, to avoid additional regulatory and interfering effects of the core circadian clock and endocrine hormones on metabolism.

In this study, we constructed the Per-KD subcultured cell line, in which PER protein expression was continuously knocked down, based on the continuous expression of shRNA (short hairpin RNA) by a vector, and observed a stable effect of the circadian clock system on cell growth and development. This result would be difficult to determine by short-term knockdown of the *Per* gene by treatment with double-stranded RNA or siRNA. Furthermore, 8 biological repeats showed almost the consistent metabolism change, making the evidence for the impact on metabolic pathways more convincing. The results of this study showed that the activities of key enzymes in glycolysis and the TCA cycle, as well as transcription levels of their coding genes, were almost consistently repressed (Fig. 6), suggesting that after knockdown of BmPER, the peripheral clock regulated glucose metabolism by affecting the expression and/or activities of certain key enzymes in glucose metabolism. Comparison of circadian transcription in zebrafish^1^, flies^48^, and mammals^37^ suggests that circadian control of several key processes and pathways have been conserved among organisms and cultured cells *in vitro*.

*Per* gene have important biological significance. It has been reported that RNA interference of the *SpPer* gene caused a significant delay in the UVD lumen acidification^49^ and affected the rhythm of sperm release in *Spodoptera littoralis* moths^50^. Also, the temporal expression of the *yolk protein 2 (yp2*) gene at the mRNA and protein level in the testis of *Spodoptera littoralis*, is regulated by *SpPer*-based molecular oscillator^51^. Sandrelli *et al*.^52^ found that *BmPer* silencing produced a small but detectable disruption in the egg-hatching rhythm, as well as a reduction in egg-to-adult developmental time. In the research of *Antheraea pernyi*, the close anatomical location between PTTH- and PER-expressing cells suggested routes of communication between these two cell populations that may be important for the circadian control of PTTH release^53^.

The protein of PER binds to nuclear receptors such as PPARα and REV-ERBα to provide another mode of circadian regulation via nuclear receptor function, which regulates numerous life activities including growth, development, endocrine activity, reproduction, and energy metabolism^19^,^22^,^54^. This binding regulation is particularly important for liver glucose metabolism^19^. Previous studies have also demonstrated that post-translational modifications may play central roles in many biological pathways, including circadian regulation^48^,^55^,^56^. The current results regarding the regulation of pyruvate synthesis showed that PK activity increased in Per-KD cells, despite a significant decrease in gene transcription and pyruvate content.

It is generally known that transcription regulation is a basic to regulate the enzyme activity^57^,^58^. In our manuscript, even though the mRNA level of *BmPk* gene down-regulated, BmPK activity upregulated in Per-KD cells. Meanwhile, content of pyruvic acid, the catalysate of BmPK, was reduced. It was reported that, the phosphorylation level of rate-limiting enzymes such as BmPK is one of the important ways to regulate glycometabolism^59^. It has been reported that excess alanine which is generated by pyruvate acid, would feedback inhibited the activity of BmPK by phosphorylation^60^,^61^. In our result, the content of alanine and pyruvate acid both decreased after *BmPer* knockdown. Therefore, we speculated that the feedback regulation promoted to dephosphorylation. This kind of post-translational regulation that caused inconsistent of transcription level and enzyme activity has already reported in *Bombyx mori* eggs^62^. However, the pyruvate content catalyzed by PK was not restored because of a decrease in metabolic level of the early glycolytic pathway, suggesting that the circadian clock may regulate cell metabolism via complex mechanisms.

The circadian clock and metabolic processes are interactive^63^. Changes in the alcohol metabolic pathway were observed in a *Drosophila Per* mutant, resulting in differences in alcohol tolerance and clearance capability^64^. On the other hand, long-term alcohol intake in mice can lead to changes in the circadian rhythm^65^, whilst damaging the normal rhythm of liver glycogen metabolism^66^. Numerous studies have reported such reciprocal regulation of the circadian clock by metabolites, whereby the metabolites regulate expression of core circadian clock genes, thus changing the circadian rhythm. For example, glucose levels can affect the expression of *mPer2* in mammalian hypothalamic cells, thus regulating the circadian clock by a feedback mechanism^67^. This type of regulation is not limited to glucose, and control of other dietary nutrients also changes the circadian rhythm^68^. In addition, some metabolites, such as cAMP and NAD^+^ 69,70, can act as input signals to regulate the circadian clock. This regulation can often be seen when the metabolites are also signaling factors. Because of the abnormal transcriptional change of other circadian genes, we speculated that both *BmPer* gene knockdown and the abnormal metabolism play a role in the change of circadian clock. This interaction between the circadian rhythm and metabolism opens up new areas of research.

**Figure 7:**
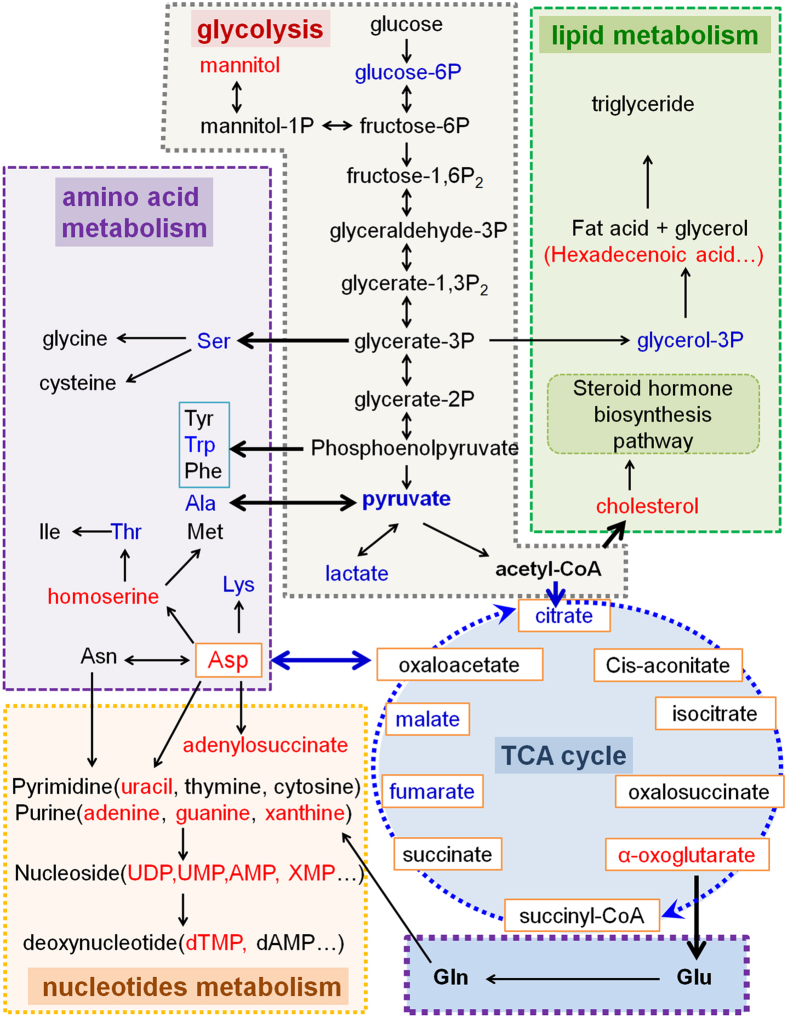
Summary of metabolic pathways affected in Per-KD cells. The pathways in the figure were simplified according to the KEGG pathways (http://www.kegg.jp/). Metabolites indicated in red were increased after knockdown of *BmPer*, while metabolites indicated in blue were decreased, as determined by metabolomics analysis. Overall glucose metabolism (including glycolysis and TCA cycle) and amino acid-like substances generated from glucose metabolic intermediates were down-regulated, while overall nucleotide metabolism was upregulated by *BmPer* knockdown.

The network of possible effects of *BmPer* gene knockdown on the metabolism of silkworm BmN cells, based on the results of metabolomic assays, is shown in Fig. 7. The glucose metabolic pathways including glycolysis and TCA cycle were decreased because of the low transcription level and activity of enzyme, and low content of metabolites. Meanwhile, the amino acids which use glucose metabolites as raw materials also decreased. For example, the Ala, who is generated by pyruvic acid, also decreased following pyruvic acid. In contrast, the impact on lipid metabolism was mainly focused on cholesterol and subsequent steroid hormone pathways, while nucleotide metabolism, including purine and pyrimidine metabolism, was increased. These results confirm the complex effect of the circadian clock system on metabolism.

Metabolic affect cell size and growth through complicated ways. Some reports showed that a mevalonate pathway regulates cell size homeostasis and proteostasis through autophagy^71^. The de novo purine synthesis in circadian controlled the cell cycle^1^. Miettinen *et al*.^72^ found that mitochondrial metabolism and lipid synthesis are used to couple cell size and cell proliferation through identification of transcriptional and metabolic programs related to mammalian cell size. Hence, the significant decrease of glycometabolism and relative amino acid metabolism level after *BmPer* knockdown might be related to cell morphologies such as small size and slow growth.

The circadian clock has been reported to affect lipid metabolism^73^, as well as having important effects on hormones and the endocrine system, mainly due to the role of the suprachiasmatic nucleus core clock^74^. Many studies have shown that steroid hormones, such as glucocorticoids^75^,^76^ and estrogen^77^, affect metabolism by regulating the circadian clock through hormone signaling pathways. The results of the current study showed that changes in the circadian clock may also affect cholesterol levels, with subsequent effects on steroid hormone pathways, suggesting that the peripheral clock may impact on hormone signaling pathways.

Few studies have reported on the effects of the circadian clock on amino acid and nucleotide metabolism. The metabolomics results in the current study showed that, when glucose metabolism was repressed, most amino acids produced from the intermediate metabolites of glucose metabolism were also reduced (Fig. 7), suggesting that regulation of amino acid metabolism by the circadian clock may be an indirect effect of its regulation of glucose metabolism. The effect of the circadian clock on nucleotides was an interesting phenomenon, involving up-regulation of pyrimidine and purine metabolism, and the subsequent formation of ribonucleotides and deoxyribonucleosides in Per-KD cells (Fig. 7). However, correlations between these nucleotide substances and other metabolites were not strong (Fig. 3), indicating that nucleotide metabolism may be a relatively independent regulatory process. Purine synthesis has been reported to be controlled by the circadian oscillation system and to affect the regulation of cell cycle by the circadian clock^1^, presumably linked to the change in growth rate of Per-KD cells in the present experiment. Further studies are needed to clarify the mechanisms whereby the circadian clock regulates nucleotide metabolism.

## Conclusion

Knockdown of the core circadian oscillation gene *BmPer* in the silkworm *B. mori* demonstrated that the circadian clock system may repress glucose metabolism by regulating the expression of key enzymes of glucose metabolism, leading to abnormalities in other metabolic systems.

## Materials and methods

### Cell lines

The *B. mori* ovary cell line (BmN) was preserved in our laboratory and cultured in Grace insect medium at 26 °C, as wild-type (WT) cells.

Pre-microRNA with mir-30 was used to construct transfection plasmid short hairpin RNAs (shRNAs). The *BmPer* cDNA sequence ^270^TATATCCATATTCTCAGGTCC^290^ was chosen for siRNAPer and inserted into shRNA to obtain shRNA-Per, which was then inserted into the pIZT/V5-His/Cat vector position between SacI^578^ and SpeI^651^ to generate the shRNA-*Per* expression vector (Figure S6). BmN cells were cultured in Grace medium containing 10% fetal bovine serum and 1% penicillin–streptomycin solution, and the medium was changed every other day. Lipofectamine LTX reagent (2 μL) and 0.5 mg of DNA plus reagent vector were diluted into 25 μL of Grace medium, incubated for 10 min, and then mixed. The mixture was added to 24-well cell culture plates seeded with BmN cells and shaken gently. At 48 h after transfection, 100 μg/mL bleomycin was added for prescreening. The medium was changed every 48 h to maintain the concentration of bleomycin in the medium. After 12 days of continuous screening, half of the total transfected cells were used for total RNA extraction. The interference efficiency of the *BmPer* gene was examined by qRT-PCR. BmN mutant cells, with stable interference of the *BmPer* gene, were identified as *BmPer* knockdown (Per-KD) cells.

Cell lines were synchronized using Dex (0.1 μM, 2 h) in darkness prior to qRT-PCR and western blotting.

### Staining

Cells were seeded into culture dishes for at least 12 h, rinsed with 1 mL phosphate-buffered saline (PBS), fixed in 4% paraformaldehyde at 25 °C for 15 min, and rinsed three times with PBST (PBS + 0.05% Tween-20). Fixed cells were treated with 0.1% Triton X-100 and rinsed three times with PBST. Cells were incubated with Dil (Beyotime, Jiangsu, China) for 1 min followed by three 5-min washes with PBST. Then, cells were incubated with 4′-6-diamidino-2-phenylindole (DAPI) (Beyotime) at room temperature for 5 min. Following three 5-min washes with PBST, the cells were observed under a fluorescence microscope (Olympus SZX16, Tokyo, Japan).

### Determination of cell growth rate

Cell growth was determined by the 3-(4,5)-dimethylthiazo (-z-y1) -3,5-diphenytetrazoliumbromide (MTT) method, according to the manufacturer’s instructions (MTT Cell Proliferation Assay Kit C0009; Beyotime). Cells were digested with trypsin and counted, and the cell solution was adjusted to 2 × 10^4^ cells/mL. Cell solution (100 μL) and 10 μL of 5 mg/mL MTT solution were then added to each well of the multi-well plate and incubated at 37 °C for 4 h, followed by the addition of 100 μL formazan solution, and incubation at 37 °C for a further 4 h. The absorbance at 570 nm was then measured using a microplate reader (Eon, Biotek, Winooski, VT, USA). The experiments were repeated three times.

### GC/LC-MS assay

Based on the previously described pretreatment method for cell metabolomics^78^, the cells were counted, 1 × 10^7^ cells were taken, and the cell supernatants were obtained by quenching and centrifugation at 1,000 *g* for 1 min.

Samples for GC-MS assay were resuspended in 500 μL of methanol pre-cooled at −80 °C, followed by the addition of 60 μL of margaric acid (0.1 mg/mL) and 60 μL of dichlorophenylalanine (0.2 mg/mL), and cryopreservation in liquid nitrogen. The samples were thawed at room temperature, resuspended in 500 μL of pre-cooled methanol, and the supernatant was obtained by centrifugation at 800 *g* for 1 min. Methanol resuspension and centrifugation were repeated. The cells were resuspended in ultra-pure water and cryopreserved in liquid nitrogen, then centrifuged at 15,000 *g* for 1 min, and dried at 30 °C in a vacuum to produce the samples to be assayed. Methoxy solution (60 μL) was added and mixed well, and the reaction was carried out at 37 °C for 2 h. Finally, 60 μL of bis-trimethylsilyl-trifluoroacetamide reagent containing 1% trimethylchlorosilane was added and the reaction took place at 37 °C for 90 min. The resulting samples were subjected to GC-MS assay (Agilent 7890A/5975C) using an HP-5MS capillary column (5% phenyl methyl silox: 30 m × 250 μm internal diameter, 0.25 μm; Agilent J & W Scientific, Folsom, CA, USA). Gas chromatographic conditions: split injection with a split ratio of 20:1, injection volume 1 μL, inlet temperature 280 °C, ion source temperature 250 °C, and interface temperature 150 °C. The initial temperature of programmed heating was 80 °C maintained for 2 min, followed by an increase to 300 °C at a rate of 10 °C/min, and then maintained for 5 min. The total operating time was 30 min, the carrier gas was helium, and the carrier gas flow rate was 1 mL/min. Mass spectrometry conditions: electrospray ionization source, full scan mode, electron energy 70 eV; quadrupole scan range 35–780 m/z.

The samples for LC-MS assay were resuspended in 500 μL of pre-cooled methanol and the supernatant was obtained by centrifugation. Methanol resuspension and centrifugation were repeated. The cells were resuspended in ultra-pure water, frozen in liquid nitrogen, and then centrifuged at 15,000 *g* for 1 min, and dried at 30 °C in a vacuum to produce the samples to be assayed. The samples were then dissolved in 300 μL of methanol solution (v:v = 1:1, 4 °C), filtered through a 0.22-μm membrane, and then subjected to LC-MS assay (Waters UPLC, Waters, Milford, MA, USA) using a C18 column (1.7 μm, 2.1 × 100 mm) (BEH, Waters). The mobile phase A/B was ultrapure water/acetonitrile, consisting of 0.1% (v/v) formic acid, the flow rate was 0.3 mL/min. Linear gradient elution as follows, 0 min–1 min, 2%B; 1 min–1 min, 2–50%B; 11 min–17 min, 50–98%B; 17 min–18 min, 98%B; 18 min–19 min, 98–2%B; 19 min–20 min, 2%. injection volume 2 μL, column temperature 40 °C, autosampler temperature maintained at 4 °C. Leucine enkephalin was used as lock and spray (0.4 ng/L, 0.1% formic acid acetonitrile/H_2_O 50/50). Mass spectrometry conditions: electrospray ionization source, positive and negative ionization modes. Source temperature 120 °C, desolvation temperature 350 °C, desolvation gas flow 800 L/h, and cone gas flow 50 L/h. The capillary ionization voltages of positive and negative ion mode were 3.0 and 2.8 kV, respectively, sampling cone 30 eV, extraction cone 4 eV, and quadrupole scan range 50–1,000 m/z. After the end of the test, the metabolites were confirmed based on their exact molecular weights and the possible empirical formulae of the metabolites were speculated (molecular weight error <30 ppm). The exact molecular weights and MS/MS fragmentation patterns were then used to identify potential biomarkers by confirmation in the Human Metabolome Database (HMDB) (http://www.hmdb.ca website), Metlin (http://metlin.scripps.edu/website), massbank (http://www.massbank.jp/), and LipidMaps (http://www.lipidmaps.org).

Each sample was extracted 20 μL and mixed as QC sample, which is used to correct the deviation of results and the errors caused by the instrument reason (Figure S7). GC-MS and LC-MS assay were carried out at BioNovoGene Co., Ltd. (Suzhou, China).

### qRT-PCR

Total RNA was isolated from WT and Per-KD BmN cells for qRT-PCR analysis using RNAiso™ Plus (TaKaRa, Dalian, China). The concentrations of RNA were determined and the qualities were controlled spectrophotometrically by the A260/A280 ratio (Beckman, Brea, CA, USA). Any DNA in the total RNA samples was digested with RNase-free DNase I (TaKaRa), and 1 μg of the total RNA was used to synthesize first-strand cDNA. qRT-PCR was carried out in a total reaction volume of 20 μL using an ABI StepOnePlus™ PCR system (Ambion, Foster City, CA, USA) and the fluorescent dye SYBR Premix Ex Taq (TaKaRa), according to the manufacturers’ instructions. The reaction conditions were as follows: 95 °C for 30 s, followed by 40 cycles at 95 °C for 5 s then 60 °C for 30 s. After PCR, melting curve analysis was used to confirm the amplification of specific products and the data were normalized against endogenous *BmRp49*^62^,^79^. The standard curve method was used to determine the expression levels of the samples. All the experiments were performed in triplicate. The gene-specific primers used in this study are given in Table S1.

### Determination of enzyme activity

Soluble protein was isolated from WT and Per-KD cells for enzyme assays. Protein concentrations were measured with a BCA kit (Beyotime), and enzyme activities were determined with an Enzyme Activity Assay Kit (Jiancheng, Nanjing, China), according to the manufacturer’s instructions. The activities of hexokinase (HK), phosphofructokinase (PFK), and pyruvate kinase (PK) were determined by measuring changes in NADPH using glucose, fructose-6-phosphate, and phosphoenolpyruvate as substrates. The activity of citrate synthase (CS) was determined by measuring citrate production with acetyl-CoA and oxaloacetate as substrates, using the dithio-bis-nitrobenzoic acid colorimetric method. One unit of HK, PFK, and PK, respectively, was defined as the consumption of 1 nM NADH/min/mg of cell protein (U/mg protein). One unit of CS was defined as the production of 1 μM citrate/min/mg of cell protein (U/mg protein).

### Western blotting

Proteins were extracted from WT and Per-KD cells and subjected to 12% sodium dodecyl sulfate-polyacrylamide gel electrophoresis and electrotransferred to a polyvinylidene difluoride membrane. The membrane was blocked with a blocking solution, followed by incubation with the specific antibody, and washed and incubated with horseradish peroxidase (HRP)-labelled anti-rabbit immunoglobulin G (Bioworld Technology, Minneapolis, MN, USA). Immunoblots were visualized using Clarity Western ECL Substrate and ChemiDoc Touch Imaging System (Bio-rad, Hercules, CA, USA). Protein levels were measured by placing a rectangle of the same size over each Per-KD and WT.

The amino acid sequence of the BmPER protein was acquired from NCBI (GenBank accession number NP_001036975). After peptide sequence design, synthesis and purification, the peptide sequence of the PER protein, NH_2_-ESGESKKRDTRNHCCONH_2_, was used as an antigen to immunize New Zealand rabbits. Antibody titers were detected by enzyme-linked immunosorbent assay. The antibodies were purified by affinity purification. All these procedures were completed by Abgent Biotechnology Co., Ltd (Suzhou, China). Antibody specificity was analyzed by western blotting.

### Statistical analysis

The original GC-MS and LC-MS data were processed and analyzed using XCMS software (www.bioconductor.org/). GC-MS and LC-MS assay results were used to identify differential metabolites between the WT and Per-KD cells using partial least squares-discriminant analysis (PLS-DA) first principal component variable importance in projection (VIP) value (VIP ≥1) and Student’s *t*-tests (p < 0.05). Statistical tests were performed using R 3.0.3 software (www.r-project.org). PCA was used for unsupervised data analysis, PLS-DA was used for supervised data analysis, and S-plot analysis was performed with Simca-P 13.0 (Umetrics AB, Umea, Sweden)^80^. The Pheatmap function of the R language package was used for Heatmap analysis (www.r-project.org).

Metabolite correlation analysis was assessed using Pearson’s correlation coefficients. The computing method was the cor() function in the R language package (www.r-project.org), and metabolite correlation analysis and statistical significance tests were performed at the same time. Statistical tests were carried out using the cor.test() function in the R language package. A false positive check was performed on the p value, and the correlation was considered to be significant at a false discovery rate p value ≤ 0.05. The relationships among metabolites for constructing the metabolic pathways were based on the Kyoto Encyclopedia of Genes and Genomes (KEGG) database^81^ (http://www.genome.jp/kegg/). Univariate analysis of variance (ANOVA) was used to determine the significance of differences in relative contents between different groups. Metabolic pathways of differential metabolites were subjected to MetPA analysis^33^ using the analysis website http://www.metaboanalyst.ca/, and the analysis parameters were the default values.

Enzyme activity and gene expression levels were analyzed by univariate ANOVA.

## Additional Information

**How to cite this article**: Tao, H. *et al*. Inhibition of expression of the circadian clock gene *Period* causes metabolic abnormalities including repression of glycometabolism in *Bombyx mori* cells. *Sci. Rep.* **7**, 46258; doi: 10.1038/srep46258 (2017).

**Publisher's note:** Springer Nature remains neutral with regard to jurisdictional claims in published maps and institutional affiliations.

## Supplementary Material

Supplementary Information

Original data for figure 4

Original data for figure 5

## Figures and Tables

**Table 1:** Differential metabolites identified by GC-MS assay.

Metabolite	RI	Q.ion.mz.	RT.s.	vip	p value	q value	fold_change
2-Hydroxyglutaric acid	1588.9	129.0	802.6	1.57	4.79E-13	4.50E-11	−2.63
Pyruvic acid	1070.7	174.0	377.4	1.57	1.25E-12	1.16E-10	−1.51
Citric acid	1843.5	273.1	970.8	1.50	4.92E-08	4.18E-06	−1.43
4-Hydroxybutanoic acid	1245.7	233.1	534.9	1.55	2.14E-10	1.90E-08	−0.84
Oxalic acid	1147.6	45.0	446.4	1.57	7.88E-12	7.17E-10	−0.80
Aconitic acid	1760.9	229.1	918.4	1.40	7.89E-06	6.23E-04	−0.75
Glyceric acid-3-phosphate*	1833.5	357.1	964.4	1.15	1.57E-03	9.54E-02	−0.60
Uric acid*	2130.3	441.2	1137.0	1.44	1.36E-06	1.10E-04	−0.56
Lactic acid	1083.1	191.0	388.3	1.57	1.55E-12	1.43E-10	−0.54
Glucose-6-phosphate*	2371.2	205.0	1260.6	1.09	3.46E-03	1.97E-01	−0.54
2-methyl-Fumaric acid	1361.9	184.0	631.6	1.16	1.31E-03	8.12E-02	−0.54
Inosine	2604.6	230.1	1371.0	1.33	6.25E-05	4.69E-03	−0.52
Tryptophan*	2227.6	202.1	1188.4	1.43	2.57E-06	2.06E-04	−0.52
Lysine*	1865.7	174.1	984.3	1.29	1.34E-04	9.24E-03	−0.43
3-Hydroxypropanoic acid	1158.4	177.0	457.7	1.46	7.21E-07	5.99E-05	−0.31
Threitol*	1529.4	103.0	760.0	1.36	2.59E-05	1.99E-03	−0.28
Threonic acid*	1565.6	292.1	786.3	1.13	2.05E-03	1.23E-01	−0.23
Alanine*	1122.7	116.1	424.0	1.31	8.47E-05	6.18E-03	−0.17
Serine*	1379.6	204.1	644.1	1.29	1.43E-04	9.75E-03	−0.12
Malic acid*	1505.8	233.1	742.3	1.31	9.55E-05	6.87E-03	−0.12
Threonine*	1403.3	117.0	663.9	1.22	4.97E-04	3.23E-02	−0.06
Adenine	1882.3	264.1	994.6	1.37	1.62E-05	1.27E-03	0.24
Guanosine	2857.1	324.1	1461.5	1.07	4.21E-03	2.32E-01	0.25
Phosphoric acid	1292.4	299.1	574.1	1.12	2.30E-03	1.36E-01	0.25
Gluconic acid	2004.4	333.1	1066.6	1.19	9.21E-04	5.89E-02	0.29
Homoserine	1463.5	183.0	711.4	1.27	2.11E-04	1.41E-02	0.33
Nicotinic acid	1302.5	180.0	583.5	1.30	1.12E-04	7.96E-03	0.35
Ketoglutaric acid*	1811.4	292.1	950.6	1.31	8.24E-05	6.10E-03	0.39
1,6-Anhydro-beta-D-glucose	1727.1	217.1	896.1	1.27	2.13E-04	1.41E-02	0.43
Cholesterol	3183.6	129.0	1633.2	1.18	1.01E-03	6.38E-02	0.48
9-(Z)-Hexadecenoic acid	2027.9	311.2	1079.9	1.53	4.99E-09	4.29E-07	0.48
Adenosine	2666.9	236.1	1398.8	1.45	9.45E-07	7.75E-05	0.51
Mannitol*	1975	205.1	1048.8	1.53	2.20E-09	1.91E-07	0.61
Monomethylphosphate	1193.0	241.0	489.3	1.30	1.21E-04	8.47E-03	0.71
Adenosine-5-monophosphate	3112.8	230.1	1594.3	1.34	4.72E-05	3.59E-03	0.76
Uracil	1350.7	241.0	622.9	1.56	1.19E-11	1.07E-09	0.82
Cysteine*	1570.3	220.1	789.5	1.54	1.29E-09	1.14E-07	0.99
N,N-Di-(2-Hydroxyethyl)-methanamine	1433.1	160.0	690.1	1.11	2.78E-03	1.61E-01	1.09
Aspartic acid*	1535.0	232.1	765.1	1.58	8.32E-14	7.91E-12	1.15
Inositol-1-phosphate	2468.5	318.1	1308.1	1.46	5.76E-07	4.83E-05	1.45
Malonic acid	1174.4	191.0	475.0	1.07	4.14E-03	2.32E-01	2.59

**Note:** RI, retention index; Q.ion.mz, quantitative ion; RT.s, retention time/second; vip, PLS-DA first principal component variable importance in projection; pvalue, *t*-test significance; Qvalue, false-positive correction of *t*-test significance; fold_change, logarithm of Per-KD/WT fold change (fold change >0 means WT > Per-KD; fold change < 0 means WT < Per-KD). Metabolites with “*” were verified by standards.

**Table 2:** Differential metabolites identified by LC-MS secondary mass spectrometry.

Metabolin	Type	Mass	RT.s	vip	p corr	p value	fold_change
Glutathione	[M + H]+	307.1	57.5	8.4	−0.91	2.74E-06	−1.03
cGMP	[M − H]−	345.0	58.0	1.4	−0.89	5.71E-04	−0.86
5-phosphoribosyl-1-diphosphate	[M − H]−	390.0	35.1	2.6	−0.85	2.40E-03	−0.62
dTMP	[M − H]−	322.1	70.4	2.0	0.88	8.13E-04	0.70
Cysteine	[M + H]+	121.0	59.1	2.2	0.88	1.16E-08	0.94
Adenine	[M + H]+	135.1	195.9	3.2	0.89	1.07E-05	1.01
2-Chloroadenosine	[M + H]+	301.1	409.6	5.5	0.81	1.58E-06	1.07
Xanthine	[M + H]+	152.0	75.5	3.0	0.90	3.31E-06	1.10
Uridine 5-diphosphoglucuronic acid	[M − H]−	580.0	48.5	1.6	0.92	9.11E-04	1.19
Xanthosine	[M + H]+	284.1	171.4	1.3	0.89	5.76E-09	1.22
Uridine monophosphate (UMP)	[M − H]−	324.0	46.4	10.4	0.98	2.62E-06	1.32
Adenylosuccinate	[M − H]−	463.1	126.0	15.3	0.96	1.07E-04	1.35

Type, ionization mode; [M + H]+, positive ion mode; [M − H]−, negative ion mode; Mass, exact molecular weight; RT.s, retention time/second; vip, PLS-DA first principal component variable importance in projection; pvalue, *t*-test significance; Pcorr, p (corr) value of S-plot correlation; fold_change, logarithm of Per-KD/WT fold change (fold change >0 means WT > Per-KD; fold change <0 means WT < Per-KD). All the metabolites listed here did MS/MS.
