# Supplementary material for: Inhibition of expression of the circadian clock gene Period causes metabolic abnormalities including repression of glycometabolism in Bombyx mori cells
Source: Sci Rep. 2017 Apr 10;7:46258. doi: 10.1038/srep46258 (PMC5385517; doi:10.1038/srep46258)
Supplement: Supplementary Information [file srep46258-s1.pdf]

Supplementary Information

Inhibition of expression of the circadian clock gene *Period* causes metabolic abnormalities including repression of glycometabolism in *Bombyx mori* cells

Hui Tao[1,2], Xue Li[1,2], Jian-Feng Qiu[1,2], Wen-Zhao Cui [1,2], Yang-Hu Sima[1,2,3], Shi-Qing Xu[1,2,3]

1 School of Biology and Basic Medical Sciences, Medical College, Soochow University, Suzhou 215123, China. 2 Institute of Agricultural Biotechnology & Ecology (IABE), Soochow University, Suzhou 215123, China. 3 National Engineering Laboratory for Modern Silk (NEAER), Soochow University, Suzhou 215123, China. Hui Tao and Xue Li contributed equally to this work. Correspondence and requests for materials should be addressed to S.-Q.X. (email: szsqxu@suda.edu.cn).

Table of Contents:

Figure S1 evolutionary tree of *Per* gene in different species.

Figure S2 Transcripts of clock genes analyzed by qRT-PCR with *BmRp49* as an internal control.

Figure S3 Total ion current GC-MS chromatogram.

Figure S4 Total ion current LC-MS chromatograms.

Figure S5 Score of partial least square discriminant analysis (PLS-DA) (a) and cluster analysis (b).

Figure S6 Vector construction.

Figure S7 the QC sample and run order in GC/LC-MS.

Table S1. Primers used in this study.

Supplementary Figures

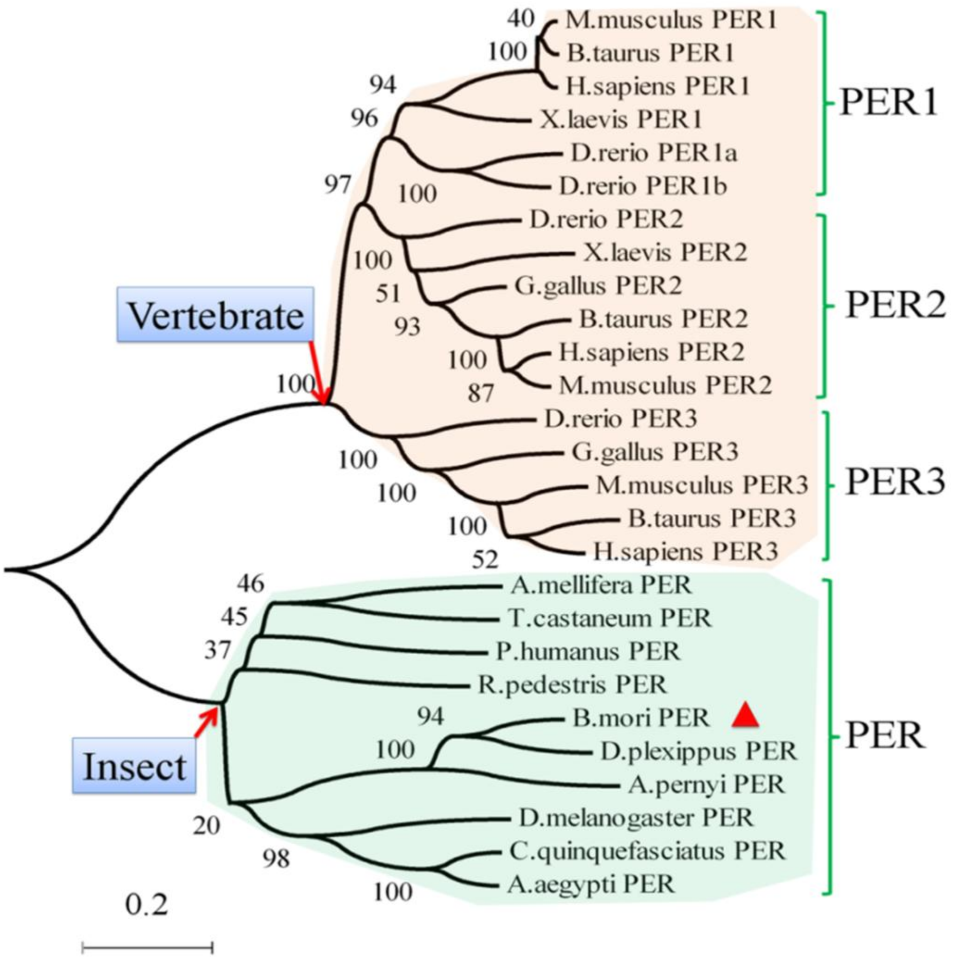

Figure S1 evolutionary tree of *Per* gene in different species. NCBI database were searched and found that vertebrate (such as *M. musculus*, *B. taurus*, *H. sapiens*, *X. laevis*) all have three types of PER (PER1, PER2, PER3), and among them, the zebra fish has two kinds of PER1 (PER1a, PER1b). But in insects such as *B. mori*, *A. penyi*, *D. plexippus* and *D. melanogaster* only have one type of PER. the evolutionary tree showed that three kind of PER in vertebrate classified into the same cluster but also distinguished clearly with each other. The only one type of PER in insects also classified into one cluster which is distinguished clearly with vertebrate. All these evidence showed that *Bombyx mori*, which is like other insects, has only one type of *Per* gene.

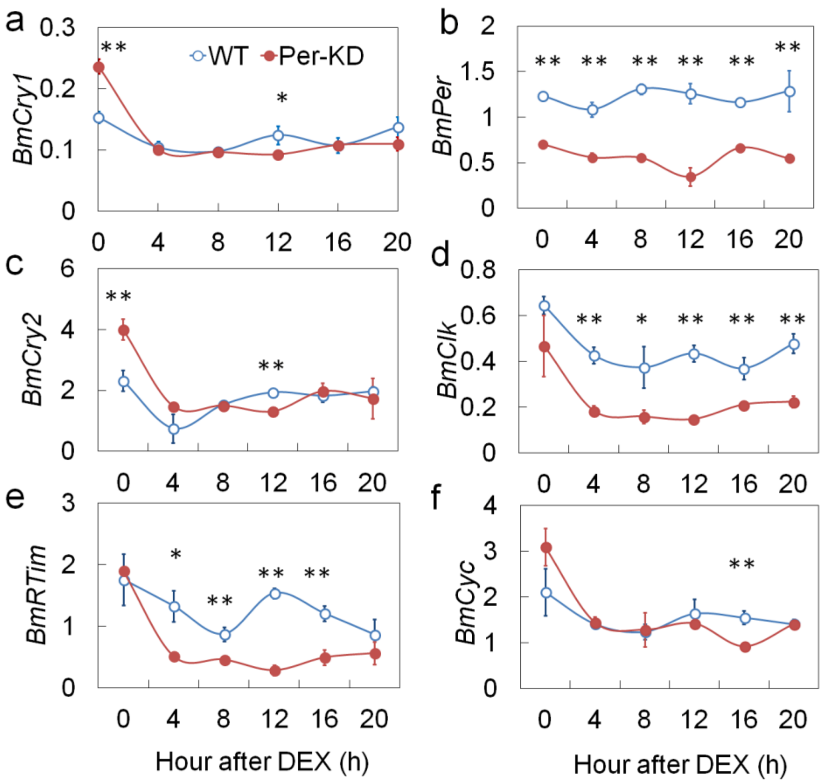

Figure S2 Transcripts of clock genes analyzed by qRT-PCR with *BmRp49* as an internal control. (a) *BmCry1*, (c) *BmPer*, (c) *BmCry2*, (d) *BmClk*, (e) *BmTim* and (f) *BmCyc* were induced in Per-KD or WT cells after synchronization for 2 h with 0.1 μM dexamethasone (Dex), and then sampled every 4 h. Per-KD, *BmPer* knockdown BmN cells; WT, wild-type BmN cells. *p ≤ 0.05 and ** p ≤ 0.01 (repeated three times).

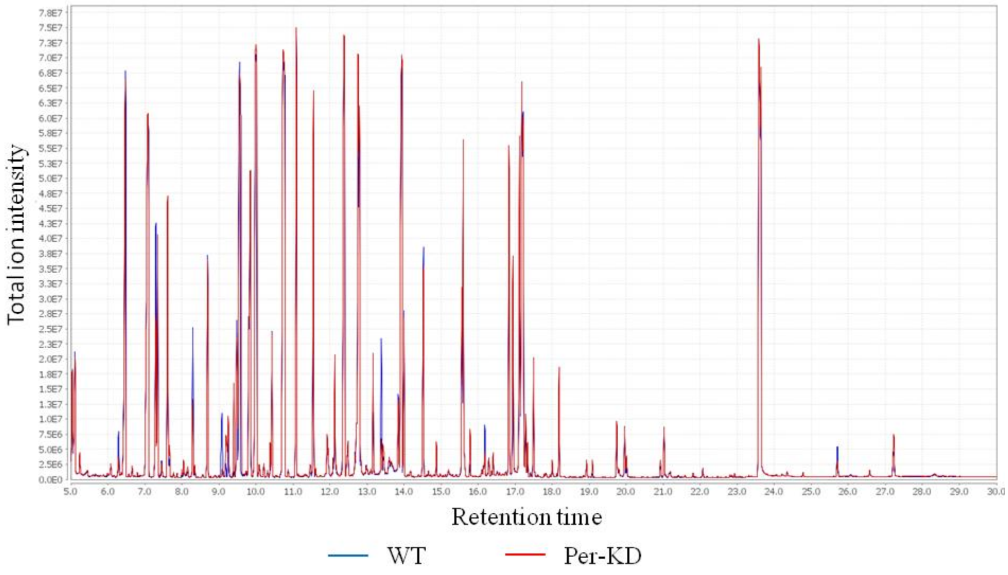

Figure S3 Total ion current GC-MS chromatogram. Gas chromatographic conditions: split injection with a split ratio of 20:1, injection volume 1 μL, inlet temperature 280 ℃, ion source temperature 250 ℃, and interface temperature 150 ℃. The initial temperature of programmed heating was 80 ℃ maintained for 2 min, followed by an increase to 300 ℃ at a rate of 10 ℃/min, and then maintained for 5 min. The total operating time was 30 min, the carrier gas was helium, and the carrier gas flow rate was 1 mL/min. Mass spectrometry conditions: electrospray ionization source, full scan mode, electron energy 70eV; quadrupole scan range 35–780 m/z.

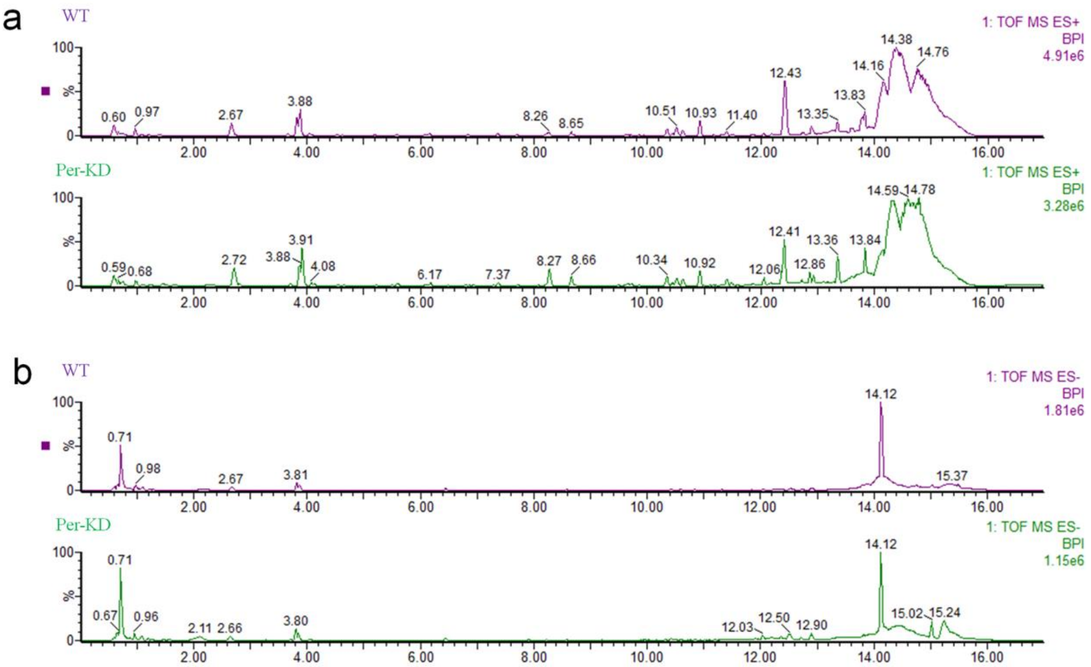

Figure S4 Total ion current LC-MS chromatograms. (a) Positive ion mode; (b) negative ion mode. The mobile phase A/B was ultrapure water/acetonitrile, consisting of 0.1% (v/v) formic acid, the flow rate was 0.3 mL/min. Linear gradient elution, injection volume 2 μL, column temperature 40 ℃, autosampler temperature maintained at 4 ℃. Leucine enkephalin was used as lock and spray (0.4 ng/l, 0.1% formic acid acetonitrile/H_2O 50/50). Mass spectrometry conditions: electrospray ionization source, positive and negative ionization modes. Source temperature 120 ℃, desolvation temperature 350 ℃, desolvation gas flow 800 L/h, and cone gas flow 50 L/h. The capillary ionization voltages of positive and negative ion mode were 3.0 and 2.8 kV, respectively, sampling cone 30 eV, extraction cone 4 eV, and quadrupole scan range 50–1,000 m/z.

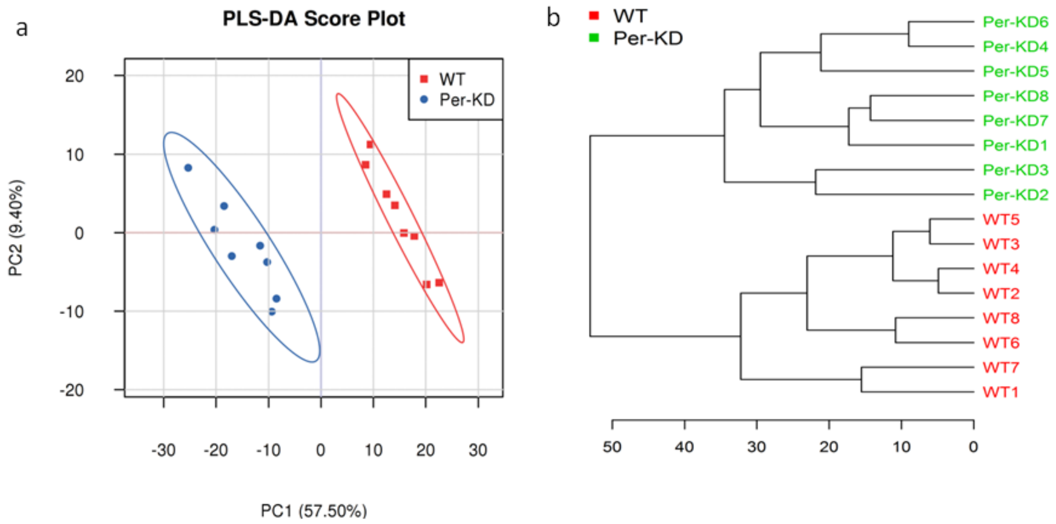

Figure S5 Score of partial least square discriminant analysis (PLS-DA) (a) and cluster analysis (b). PLS-DA data were analyzed using Simca-P 13.0 (Umetrics AB, Umea, Sweden), while tree clustering was based on Euclidean clustering and the greatest distance method.

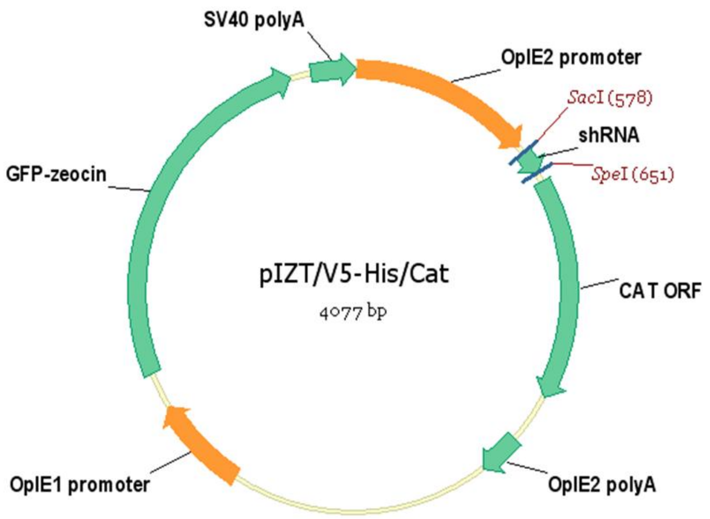

Figure S6 Vector construction. shRNA_*Per* (GCGAC**TATATCCATATTCTCAGGTCC**CT GTGAAGCCACAGATGGG**GGACCTGAGAATATGGATATA**GCTGC) was inserted into the universal vector pIZT/V5-His/Cat between SacI (position 578) and SpeI (position 651) to generate a plasmid vector capable of expressing a sequence to silence *Per*, using the OpIE2 promoter, which can be expressed in silkworm cells.

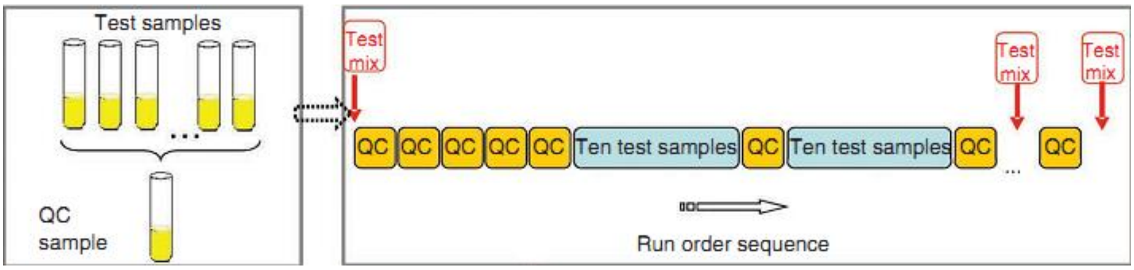

Figure S7 The quality control (QC) sample and run order in GC/LC-MS

Supplementary Tables

Table S1 Primers used in this study

Primer name	Sequence
Ribosomal protein 49 *(Rp49)*	S: CAGGCGGTTCAAGGGTCAATAC<br>A: TGCTGGGCTCTTTCCACGA
Hexokinase *(Hk)*	S: ATGCTATGCGGCTCGAC<br>A: TGTACCACCCAAATCCAA
6-Phosphofructokinase *(6Pfk)*	S: CTTCATTGGCTCGTTGAG<br>A: CATTGCTGTTTTGCTATTCT
Pyruvate kinase *(Pk)*	S: CCATCGCATTGGACACTA<br>A: CCTGGCTTCACGACATTC
Period *(Per)*	S: GAAACGGAAACTGTATCGC<br>A: GAGGCAACAGAAGTAGTCA
